# In Vitro Anti-Hepatitis B Virus Activity of Hydroxytyrosol from *Lindernia ruellioides*

**DOI:** 10.3390/molecules30092063

**Published:** 2025-05-06

**Authors:** Tong-Shi-Yao Zhao, Kang-Zhi Li, He-Ling Su, Bin Liang, Cheng-Qin Liang, Jin-Tao Gao, Xian-Li Zhou

**Affiliations:** 1 Key Laboratory of Molecular Medical Engineering, Education Department of Guangxi Zhuang Autonomous Region, Guilin Medical University, Guilin 541100, China; tsy.zhao@outlook.com (T.-S.-Y.Z.); kk106@glmc.edu.cn (K.-Z.L.); helingsu@glmc.edu.cn (H.-L.S.); bliang2022@glmc.edu.cn (B.L.); 2 College of Pharmacy, Guilin Medical University, Guilin 541100, China; cqliang@glmc.edu.cn; 3 Key Laboratory of Medical Biotechnology and Translational Medicine, Education Department of Guangxi Zhuang Autonomous Region, Guilin Medical University, Guilin 541100, China

**Keywords:** *Lindernia ruellioides*, hydroxytyrosol, anti-hepatitis B virus

## Abstract

Hepatitis B is a serious infectious disease that threatens the health of all mankind. In this study, we isolated and extracted hydroxytyrosol from *Lindernia ruellioides* with anti-hepatitis B virus (HBV) activity. The structure of hydroxytyrosol was identified by the nuclear magnetic resonance technique. HepG2.2.15 cell models were used to detect the anti-HBV activity and liver protection of hydroxytyrosol in vitro. Hydroxytyrosol can inhibit hepatitis B surface antigen (HBsAg) and hepatitis B e-antigen (HBeAg). The IC_50_ values of HBsAg and HBeAg were 4.02 mg/L and 5.19 mg/L, respectively. At the highest concentration of hydroxytyrosol, the inhibition rates of supernatant and intracellular HBV DNA were 75.99% and 66.33%, respectively. Hydroxytyrosol was less toxic to normal human hepatocytes. Molecular docking showed that hydroxytyrosol was bound to three amino acid residues of HBV polymerase with a binding energy of −7.0 kcal/mol. This study provided data for the development and utilization of *Lindernia ruellioides* and the research and development of anti-hepatitis B virus drugs.

## 1. Introduction

Hepatitis B is one of the most prevalent infectious diseases globally, which can lead to both acute and chronic hepatitis; approximately 254 million individuals worldwide are currently infected [1,2]. In 2022, hepatitis B caused an estimated 1,100,000 deaths [1]. The main causes of death were cirrhosis and hepatocellular carcinoma [1]. HBV is a DNA virus that produces several antigens critical for the diagnosis of HBV infection, including the surface antigen (HBsAg), core antigen (HBcAg), and pre-core antigen (HBeAg) [3]. These antigens serve as key biomarkers for detecting and monitoring HBV infection. The limitations of commonly used therapy for HBV are becoming increasingly apparent [4]. Currently approved anti-HBV drugs are pegylated interferon alpha (PEG-IFN-alpha) and nucleoside analogs (NAs) [5,6]. Interferon is an immune modulator of antiviral immune response, but the response rate is low (≤ 20%) and can cause a variety of side effects [7]. Nucleosides only inhibit the later stage of the HBV life cycle (reverse transcriptional step), and because they cannot target cccDNA, long-term medication is required [8]. Given these challenges, developing novel antiviral drugs is essential for future anti-HBV treatment.

As an important source of novel biomolecules, natural products and their derivatives hold broad therapeutic potential. A significant number of modern medicines are derived from medicinal herbs and used to treat various diseases [9,10,11]. As an alternative to conventional chemical drugs, many Chinese herbs and their related active substances have been reported to have good ability to control chronic HBV infection, such as swertia [12], Artemisia flavescens [13], matrine [14,15], etc. These compounds offer several advantages, such as structural diversity, safety, and the ability to block different stages of the virus’s life cycle [15,16]. Additionally, they reduce the likelihood of drug resistance development [15,16].

*Lindernia ruellioides* is the whole grass of *Lindernia ruellioides* (*Colsm.*) *Pennell*, an annual herb belonging to the Scrophulariaceae family [17]. It is a commonly used folk medicine, which has the effects of reducing swelling, relieving pain and promoting blood circulation. It is often used in folk medicine to treat bruises, snake and dog bites, menstrual irregularities, and other diseases [16,18]. Hydroxytyrosol is a polyphenol isolated from *L. ruellioides*. Hydroxytyrosol has been shown to possess antibacterial, antioxidant, anti-inflammatory, anti-tumor, antiviral, and other pharmacological activities [19,20,21]. Previous studies have found that phenylethanol glycosides of *Lindernia ruellioides* had an anti-hepatitis B effect [22]. In this study, hydroxytyrosol was isolated from *Lindernia ruellioides* and tested on the HepG2.2.15 cell line to evaluate its antiviral effects in vitro to develop a novel anti-HBV drug.

## 2. Results

### 2.1. Structural Identification

**Figure 1:**
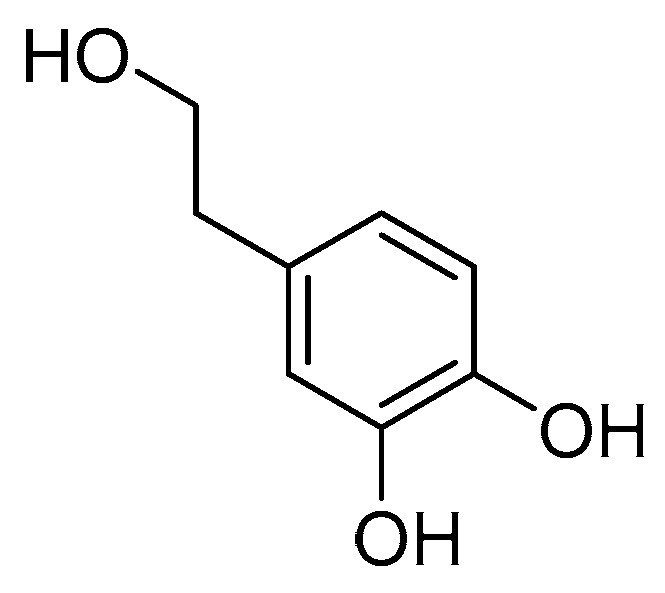
Chemical structure of hydroxytyrosol.

The compound identified as hydroxytyrosol (Figure 1) is brown and oily. ^1^H-NMR (400 MHz, CD_3_OD) δ: 6.64 (1H, d, *J* = 8.0 Hz, H-5), 6.61 (1H, d, *J* = 1.7 Hz, H-2), 6.48 (1H, dd, *J* = 8.0, 1.7 Hz, H-6), 3.63 (2H, t, *J* = 7.3 Hz, H-8), 2.62 (2H, t, *J* = 7.3 Hz, H-7); ^13^C-NMR (CD_3_OD, 100 MHz) δ: 131.7 (C-1), 116.3 (C-2), 146.1 (C-3), 144.6 (C-4), 117.1 (C-5), 121.2 (C-6), 39.6 (C-7), 64.6 (C-8). The above data are basically consistent with the literature reports [23]. The structure of the compound was further confirmed by the hydrogen spectrum and carbon spectrum (see Figures S1 and S2 in the Supplementary Materials).

### 2.2. Toxicity of Hydroxytyrosol to HepG2.2.15 Cells

**Figure 2:**
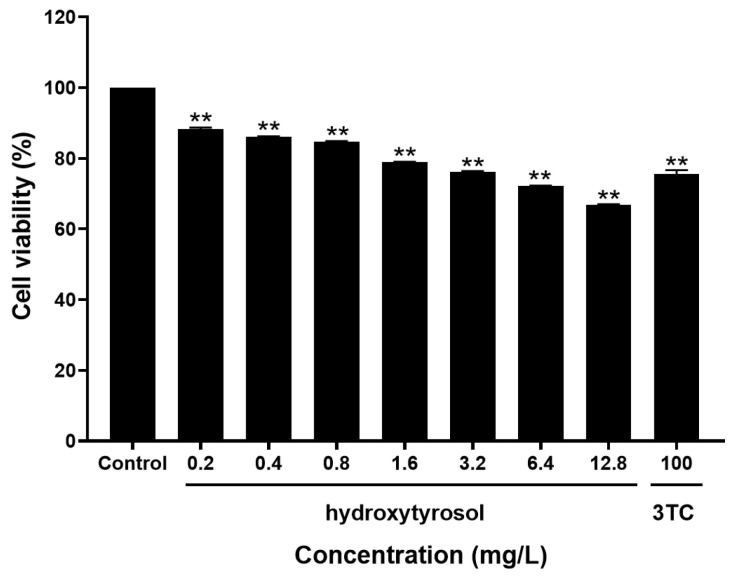
Inhibition of HepG2.15 cells by hydroxytyrosol. The inhibitory effect of hydroxytyrosol on the growth of HepG2.15 cells was determined using the MTT assay. 3TC served as the positive control. ** *p* < 0.01 vs. negative group.

As shown in Figure 2, the inhibitory effect of hydroxytyrosol on cell growth was enhanced with the increase in its concentration. The median inhibitory concentration of the tested substance on the cells was TC_50_ = 114.18 mg/L.

### 2.3. Detection of Cell Supernatant HBsAg

**Figure 3:**
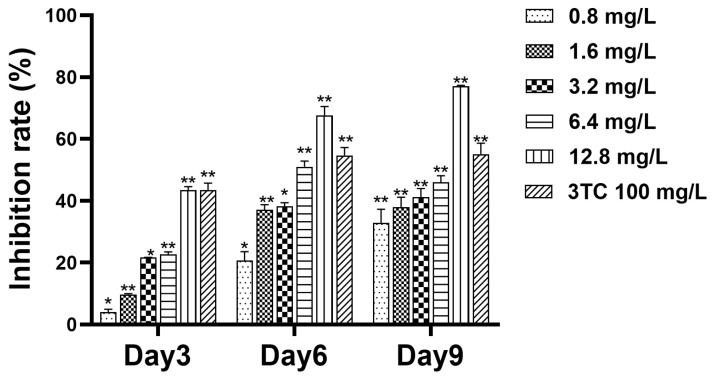
The inhibitory effects of hydroxytyrosol on supernatant HBsAg of HepG2.2.15 cells. The expression level of HBsAg was detected by ELISA. 3TC was the positive control; * *p* < 0.05, ** *p* < 0.01 vs. negative group.

In the cell experiment, HepG2.2.15 cells were treated with hydroxytyrosol. The levels of HBsAg were determined by the enzyme-linked immunosorbent assay (ELISA) method to evaluate the antiviral effect. Samples were collected from the supernatant on days 3, 6, and 9, and the levels of HBsAg were measured. The results (Figure 3) indicated that hydroxytyrosol could inhibit the secretion of HBsAg, with the inhibitory effect enhanced as the drug concentration increased. Specifically, on day 3, the inhibition rates of HBsAg increased with the concentration of hydroxytyrosol: 3.97% (0.8 mg/L), 9.71% (1.6 mg/L), 21.61% (3.2 mg/L), 22.65% (6.4 mg/L), and 43.42% (12.8 mg/L). On day 6, the inhibition rates were 20.66% (0.8 mg/L), 37.40% (1.6 mg/L), 37.48% (3.2 mg/L), 50.69% (6.4 mg/L), and 67.67% (12.8 mg/L). On day 9, the inhibition rates remained the same: 32.74% (0.8 mg/L), 37.79% (1.6 mg/L), 41.05% (3.2 mg/L), 45.97% (6.4 mg/L), and 77.02% (12.8 mg/L). The IC_50_ of day 9 was 4.02 mg/L, TI_HBsAg_ = TC_50_/IC_50_ = 28.40, TI > 2, indicating strong antiviral activity with minimal harmful effects.

### 2.4. Detection of Cell Supernatant HBeAg

**Figure 4:**
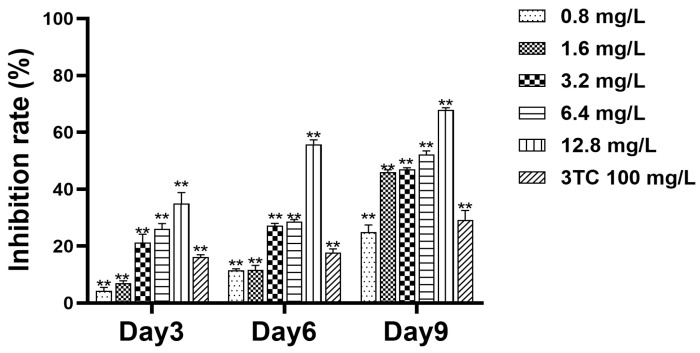
The inhibitory effects of hydroxytyrosol on supernatant HBeAg of HepG2.2.15 cells. The expression level of HBeAg in the culture medium was detected by ELISA. 3TC was the positive control; ** *p* < 0.01 vs. negative group.

HepG2.2.15 cells were treated with hydroxytyrosol. In this experiment, the levels of THBeAg were determined by the ELISA method to evaluate the antiviral effect. After the third, sixth, and ninth days, the supernatant of the cells was collected, and the expression level of HBeAg in the culture medium was detected. The results (Figure 4) showed that hydroxytyrosol could inhibit HBeAg secretion, and the inhibitory effect increased with the increase in drug concentration. On day 3, the inhibition rates of HBeAg were 4.61% (0.8 mg/L), 7.54% (1.6 mg/L), 22.75% (3.2 mg/L), 27.92% (6.4 mg/L), and 37.35% (12.8 mg/L), respectively. On day 6, the inhibition rates of HBeAg were 12.15% (0.8 mg/L), 12.32% (1.6 mg/L), 28.62% (3.2 mg/L), 30.10% (6.4 mg/L), and 58.86% (12.8 mg/L), respectively. On day 9, the inhibition rates of HBeAg were 25.35% (0.8 mg/L), 36.09% (1.6 mg/L), 47.36% (3.2 mg/L), 50.51% (6.4 mg/L), and 61.19% (12.8 mg/L), respectively (Figure 4). The IC_50_ on day 9 was 5.19 mg/L, TI_HBsAg_ = TC_50_/IC_50_ = 22.00, TI > 2, indicating that the drug is highly effective and low in toxicity.

### 2.5. Detection of Cell Supernatant HBV DNA

**Figure 5:**
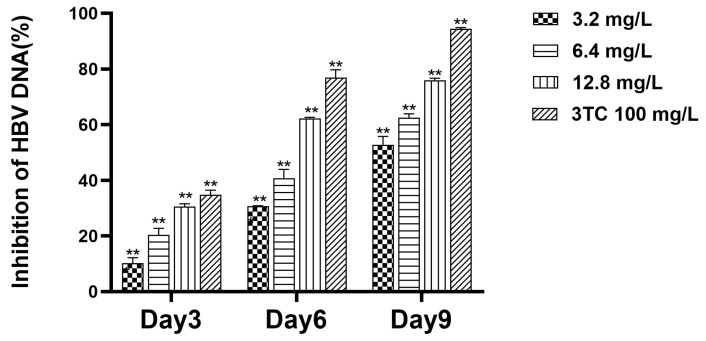
Inhibitory effect of hydroxytyrosol on HBV DNA in supernatant of HepG2.2.15 cells. The cell supernatant HBV DNA was detected by FQ-PCR. 3TC was the positive control; ** *p* < 0.01 vs. negative group.

To investigate the inhibitory effect of hydroxytyrosol on HBV DNA in the supernatant, three dosing concentrations were established, and the compound was administered every three days. Supernatant samples from the cell culture were collected on the third, sixth, and ninth days. Subsequently, the Fluorescence Quantitative Polymerase Chain Reaction (FQ-PCR) technique was used to quantitatively analyze the DNA present in the samples. The inhibition rates of hydroxytyrosol at 3.2 mg/L, 6.4 mg/L, and 12.8 mg/L on the third day were 10.29%, 20.40%, and 30.48%, respectively. On the sixth day, the inhibition rates were 30.65%, 40.58%, and 62.30%, respectively. On day 9, the inhibition rates were 52.93%, 62.53%, and 75.99%, respectively (Figure 5). The results showed that the drug had certain inhibitory effect on HBV DNA in the supernatant.

### 2.6. Detection of Intracellular HBV DNA

**Figure 6:**
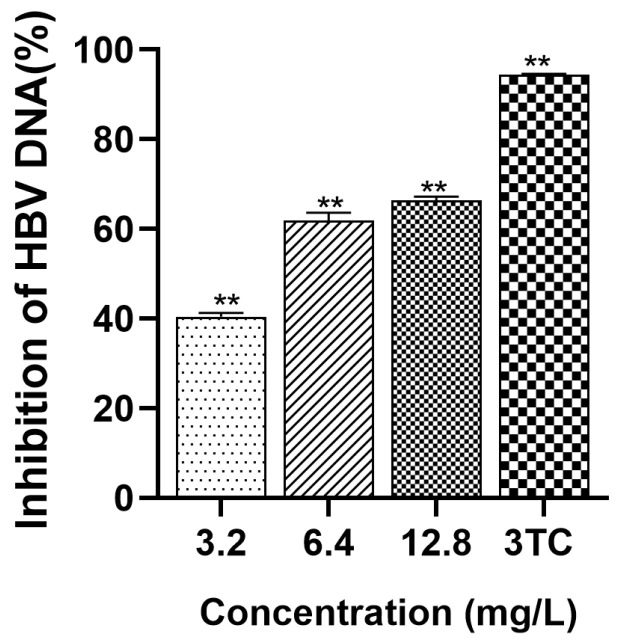
Inhibition of HBV DNA in cells by hydroxytyrosol. The intracellular supernatant HBV DNA was detected by FQ-PCR. 3TC was the positive control; ** *p* < 0.01 vs. negative group.

To explore the potential of hydroxytyrosol to curtail the levels of intracellular HBV DNA, three concentrations were set for continuous administration for 9 days, and cells were collected at the ninth stage for detection. DNA quantification was carried out using FQ-PCR. As shown in Figure 6, the intracellular inhibition rates of hydroxytyrosol on day 9 were 40.49%, 61.88%, and 66.33%, respectively, when the concentration was 3.2 mg/L, 6.4 mg/L, and 12.8 mg/L. Both compounds have a good inhibitory effect on intracellular HBV DNA.

### 2.7. Intracellular Distribution of Hydroxytyrosol

**Figure 7:**
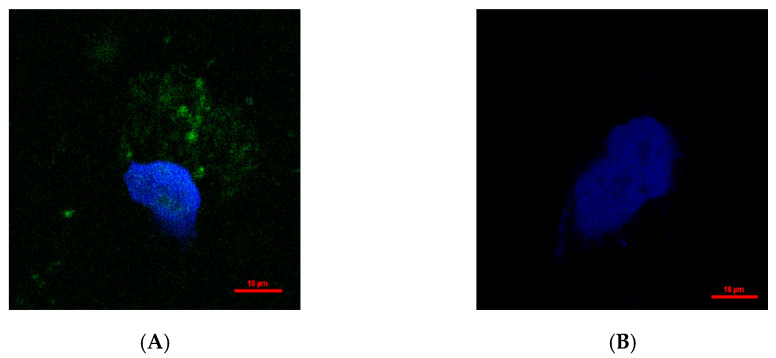
Distribution of hydroxytyrosol in HepG2.2.15 cells. There was green fluorescence in both the nucleus and protoplasm after drug action (**A**) but no green fluorescence in the negative group (**B**).

In order to further investigate the intracellular localization of hydroxytyrosol, DAPI (nuclear counterstain) and hydroxytyrosol were added to the medium where HepG2.2.15 cells were cultured, followed by observation under confocal microscopy. There was green fluorescence in both the nucleus and protoplasm after drug action, but no green fluorescence in the negative group (Figure 7). The negative control group in this experiment was composed of untreated cells, which were used as a control for assessing the background fluorescence level. As expected, no green fluorescence was detected in the negative control group. Surface drugs may bind to nucleic acid or proteins after entering the cell.

### 2.8. Study on Molecular Docking with Hepatitis B Virus Polymerase

**Figure 8:**
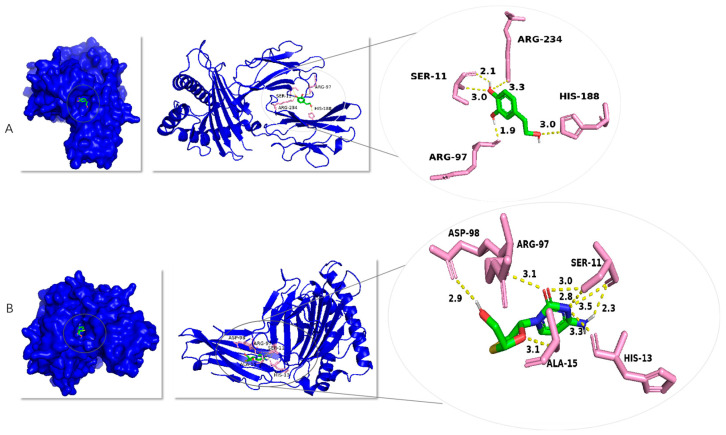
The molecular dock of hydroxytyrosol with hepatitis B virus polymerase. In the 3D structure, the hepatitis B virus polymerase is colored blue, the compound is colored with a green rod, and the docking amino acid residues are colored with a pink rod. Hydroxytyrosol and hepatitis B virus polymerase docking diagram (**A**). Lamivudine and hepatitis B virus polymerase docking diagram (**B**).

Since HBV polymerase plays a crucial role in the viral life cycle, inhibiting its activity can effectively block viral replication. Consequently, HBV polymerase has emerged as a critical target for the development of anti-hepatitis B therapeutics. This study aims to investigate the interaction between hydroxytyrosol and HBV polymerase, with the objective of exploring the potential of hydroxytyrosol as an anti-hepatitis B agent. In order to further elucidate the mechanism of interaction between active compounds and proteins at the molecular level, hydroxytyrosol was speculatively docked with hepatitis B virus polymerase. As shown in Figure 8, hydroxytyrosol can dock in the substrate binding chamber of HBV polymerase. The optimal conformation of the interaction between hydroxytyrosol and HBV RNA polymerase has a binding energy of −7.0 kcal/mol, with hydroxytyrosol specifically binding to four amino acid residues (HIS-188, ARG-97, ARG-234, and SER-11) on the hepatitis B virus polymerase and forming hydrogen bonds: the nitrogen atom of HIS-188 forms a 3.0 Å hydrogen bond with the hydroxyl oxygen atom of hydroxytyrosol, the oxygen atom of ARG-97 residue forms a 1.9 Å hydrogen bond with the hydrogen atom of hydroxytyrosol, the nitrogen atom of ARG-234 forms a 3.3 Å hydrogen bond with the phenolic hydroxyl oxygen atom of hydroxytyrosol, and the oxygen atom of SER-11 forms a 2.1 Å hydrogen bond with the hydrogen atom of hydroxytyrosol while its nitrogen atom forms a 3.0 Å hydrogen bond with the hydroxyl oxygen atom of hydroxytyrosol. The optimal conformation of the interaction between lamivudine and HBV RNA polymerase has a binding energy of −6.5 kcal/mol, with lamivudine specifically binding to five amino acid residues (ASP-98, ARG-97, ALA-15, SER-11, and HIS-13) on the hepatitis B virus polymerase and forming hydrogen bonds: the oxygen atom of ASP-98 forms a 2.9 Å hydrogen bond with the hydrogen atom of lamivudine, the nitrogen atom of ALA-15 forms a 3.1 Å hydrogen bond with the oxygen atom of lamivudine, the oxygen atom of ARG-97 forms a 3.1 Å hydrogen bond with the oxygen atom of lamivudine, the hydroxyl oxygen atom of SER-11 forms a 3.0 Å hydrogen bond with the oxygen atom of lamivudine and a 2.8 Å hydrogen bond with the nitrogen atom of lamivudine, the carbonyl oxygen atom of SER-11 forms a 3.5 Å hydrogen bond with the nitrogen atom of lamivudine and a 2.3 Å hydrogen bond with the hydrogen atom of lamivudine, and the oxygen atom of HIS-13 forms a 3.3 Å hydrogen bond with the nitrogen atom of lamivudine.

## 3. Discussion

Hepatitis B virus infection is a major cause of chronic hepatitis, cirrhosis, and hepatocellular carcinoma, while its treatment remains clinically challenging [24]. In recent years, recent studies have discovered the existence of anti-hepatitis B virus active ingredients in natural products [25]. In this study, hydroxytyrosol, a natural polyphenol compound, was isolated from *Lindernia ruellioides* [26]. The envelope of HBV is composed of small, medium, and large hepatitis B surface antigen, which surrounds hepatitis B virus polymerase (Pol), nucleocapsid, and core protein (HBc) [27]. HBV genomic DNA contains a positive and negative strand with four ORFs (C, X, P, and S). The three surface antigens come from S, the polymerase comes from P, and the e antigen comes from C. Serum HBsAg, HBeAg, and HBV DNA levels increase during the immune tolerance phase of HBV infection [28]. They begin to decline after entering the immune clearance period [28]. The current therapeutic goal is to achieve functional cure, i.e., no HBsAg and HBV DNA are detected in serum [29]. HBV surface antigen quantification has been widely used as a marker of HBV infection [30]. Consequently, HBsAg, HBeAg, and HBV DNA are important indicators for drug screening.

In this study, hydroxytyrosol demonstrated a certain inhibitory effect on HBsAg and HBeAg, and there is a dose-effect and time-dependent. On the ninth day of 12.8 mg/L, the inhibition rate of HBsAg reached 77.02%, the inhibition rate of HBeAg was 61.19%, the inhibition rate of extracellular HBV DNA was 75.99%, and the inhibition rate of intracellular HBV DNA was 66.33%. The results showed that the inhibitory effect of hydroxytyrosol on the secretion of HBsAg and HBeAg at high concentrations is significantly stronger compared to lamivudine. Collectively, these data demonstrate that hydroxytyrosol effectively suppresses both HBV antigen production and viral DNA replication.

MTT assay was used to detect cytotoxicity [31]. The results demonstrated that hydroxytyrosol exhibited low cytotoxicity toward HepG2.2.15 cells, maintaining normal cellular morphology within the selected concentration range. In addition, confocal laser microscopy revealed that hydroxytyrosol could enter the cytoplasm and nucleus of HepG2.2.15 cells, but the specific site of action remains to be elucidated.

Molecular docking is a tool for simulating the interaction between a ligand and a receptor, which can be used to screen active ingredients, evaluate the binding affinities between active ingredients and targets, and explain their pharmacological mechanisms [32]. This approach helps to accelerate research into the pharmacological mechanisms of Chinese herbal medicine and the development of new drugs [33]. HBV replication depends on reverse transcription catalyzed by polymerase protein. HBV polymerase is an enzymatically active protein that plays a central role in the process of HBV replication [34]. It is crucial for the treatment of hepatitis B, so it is an important target for the development of new drugs for HBV [35,36]. In this study, molecular docking was used to calculate the affinity and binding mode of hydroxytyrosol to HBV polymerase. Hydroxytyrosol binds to amino acid residues of HBV polymerase via hydrogen bonding. The calculated binding energy of hydroxytyrosol and HBV polymerase was −7.0 kcal/mol, indicating that the affinity between the ligand and the receptor demonstrated favorably. These results suggest that hydroxytyrosol may exert anti-HBV effects by targeting HBV polymerase.

In this study, hydroxytyrosol a polyphenol derived from *Lindernia Ruellioides*, an important folk medicinal plant, demonstrated significant anti-HBV activity in vitro, revealing its potential anti-HBV infection and providing a crucial scientific foundation for the study of anti-HBV components of *Lindernia ruellioides* [8].

## 4. Materials and Methods

### 4.1. Plant Material

*Lindernia ruellioides* was purchased from Pingnan, Guigang, Guangxi, China. The herb was identified as the whole grass of *Lindernia ruellioides (Colsm.) Pennell* by Associate Professor Deqing Huang of Guilin Medical College.

### 4.2. Cell Cultures

HepG2.2.15 cell line, a HepG2 cell line transfected with hepatitis B virus (HBV), sourced from Professor Su Heling of Guilin Medical College, was preserved in our laboratory and cultured by self-passage. The cells were cultured in DMEM medium (Gibco, Grand Island, NY, USA) with 10% fetal bovine serum (Gibco, USA) in a 5% CO_2_ incubator (Thermo Scientific, Waltham, MA, USA) at 37 °C.

### 4.3. Extraction and Isolation

*Lindernia ruellioides* (4 Kg) was crushed and extracted by reflux with 75% ethanol for 3 times, each time for 2 h to obtain extract, which was dispersed with appropriate amount of water, followed by a certain volume of ethyl acetate and reduced pressure distillation to recover the solvent and obtain ethyl acetate (78 g) extract. The extract was separated by silica gel column chromatography (100–200 mesh). Gradient elution was performed with chloroform–methanol solution (1:0~0:1), and the same flow fraction was detected by TCL and combined to obtain 6 components Fr.I~Fr.VI. Fr.II (9:1) was eluted with petroleum ether ethyl acetate (1:0~0:1) on silica gel column, and 7 Fr.II-1~Fr.II-7 were obtained. Fr.II-5 was separated by preparative reversed-phase silica gel column and eluted with water and 10%, 30%, and 50% methanol solution successively to obtain Fr.II-5.1~Fr.II-5.4. Fr.II-5.2 was further isolated and purified by high performance liquid chromatography (gradient elution of 10~30% methanol solution, Agilent, Santa Clara, CA, USA) to obtain compound hydroxytyrosol (23.4 mg).

### 4.4. The Toxicity of Hydroxytyrosol to HepG2.2.15 Cells Was Determined by MTT Assay

A cell suspension with a density of 2.3 × 10^4^ cells/mL was prepared at a dosage of 200 μL per well. A 96-well plate was taken out, and seven different concentrations were set for each drug, with three replicates for each concentration. Meanwhile, wells without drug addition were set as negative control wells, and wells without cell seeding but filled with culture medium were set as blank control wells. To avoid edge effects, the outermost wells of the plate were not seeded with cells. Twenty-four hours after seeding, the cells were treated with seven concentrations of drugs, namely, 0.2, 0.4, 0.8, 1.6, 3.2, 6.4, and 12.8 mg/L. The concentration of the positive control drug lamivudine (3TC) was set at 100 mg/L. The drugs were administered every 72 h for three consecutive times, with the entire administration period lasting 9 days. On the 9th day of administration, the supernatant in each well was carefully aspirated. Then, 20 μL of 5 mg/mL MTT solution (Solarbio, Beijing, China) and 180 μL of culture medium were added to each well. The 96-well plate was incubated in a cell culture incubator in the dark for 4 h. After incubation, the supernatant was gently removed, taking care not to disturb the formazan crystals at the bottom. These formazan crystals are the result of the reaction between MTT and mitochondrial dehydrogenases in viable cells, which reduce MTT to an insoluble formazan product. Subsequently, 150 μL of DMSO was added to each well, and the 96-well plate was shaken uniformly on a horizontal shaker for 10 min to completely dissolve the formazan crystals in all wells. Once the formazan crystals were completely dissolved, the optical absorbance of each well at 490 nm was measured using a microplate reader (Thermo Scientific, USA). The experimental results were recorded, the cell inhibition rates at various drug concentrations were calculated according to the formula, and the TC_50_ values were determined using appropriate statistical methods to evaluate the inhibitory effect of the drugs on the cells.

### 4.5. Detection of Cell Supernatant HBsAg and HBeAg

Twenty-four hours after cell seeding, the supernatant in each well was carefully aspirated and discarded. Hydroxytyrosol was then applied to the cells at concentrations of 0.8, 1.6, 3.2, 6.4, and 12.8 mg/L, respectively. The drug was administered once every three days, and a total of three administrations were carried out. On the 3rd, 6th, and 9th days after the first drug administration, the supernatant in each well was collected, respectively, and stored at −20 °C for subsequent analysis. The secretion levels of HBsAg and HBeAg in the collected supernatants were measured using an ELISA kit (Kehua, Shanghai, China).

### 4.6. Cell Supernatant HBV DNA Copy Number Was Detected by FQ-PCR

HepG2.2.15 cells with a density of 4 × 10^4^ cells/mL were thoroughly mixed and seeded into 48-well plates. Then, the plates were cultured in a CO_2_ incubator at 37 °C. When the cells reached approximately 50% confluence (determined by observing under a microscope that the cells covered about 50% of the bottom area of the culture vessel), the drugs were added. Three concentrations of the drug were set, namely, 3.2, 6.4, and 12.8 mg/L. The positive control drug, lamivudine, had a concentration of 100 mg/L. A group of wells without drug addition was set as the control group. Each concentration had three replicate wells. The drugs were administered once every three days, and a total of three administrations were carried out. On the 3rd, 6th, and 9th days after the first drug administration, the cell supernatant from each well was collected into a sterile and enzyme-free EP tube and stored at −20 °C. Hepatitis B virus nucleic acid assay kit (Daan Gene, Guangzhou, China) was used to detect HBV DNA in the cell supernatant.

HBV DNA inhibition rate (%) = (Control copy number − Number of copies in the administration group/Control copy number) × 100%(1)

### 4.7. Intracellular HBV DNA Copy Number Was Detected by FQ-PCR

On the 9th day of drug administration, the cell supernatant was carefully aspirated and discarded. Subsequently, the adherent cells at the bottom of each well were gently washed twice with phosphate-buffered saline (PBS) to remove any residual culture medium and debris. Then, trypsin solution was added to each well, and the plate was incubated at 37 °C for 3 min. After 3 min, a culture medium was added to terminate the digestion process. Next, the cell suspension in each well was carefully transferred into a sterile, enzyme-free Eppendorf (EP) tube and centrifuged for 5 min. After centrifugation, the supernatant was aspirated, and the cells were washed again with PBS. The extraction and detection of intracellular DNA were carried out following the experimental procedures described in “4.6”. The calculation method of intracellular HBV DNA inhibition rate is the same as “4.6” Formula (1).

### 4.8. Detection of Hydroxytyrosol Distribution in HepG2.2.15 Cells

Cells exhibiting excellent logarithmic growth activity were selected and seeded onto 15 mm confocal glass substrates at a density of 1 × 10^4^ cells/mL. After 24 h of incubation, 12.8 mg/L of hydroxytyrosol was added to the cell cultures. A negative control group was established simultaneously, where cells were subjected to the same procedures but without the addition of hydroxytyrosol. Following a 10 min drug treatment, the drug-containing medium was carefully aspirated and discarded. The cells were then washed three times with PBS to ensure the complete removal of any residual drug and culture medium components. Finally, the cells were observed and imaged under a confocal laser scanning microscope.

### 4.9. Molecular Docking Analysis of Hydroxytyrosol and Hepatitis B Virus RNA Polymerase

First, find the HBV RNA polymerase protein (PDB ID: 2hn7) in the PDB database (https://www.rcsb.org/ accessed on 1 February 2025) and download it as a PDB file. Then, draw the 3D structures of the two active compounds, including hydroxytyrosol and lamivudine, in Chem3D software. According to the ligand and protein-generating activity pockets, smina was used to perform molecular docking of these two active compounds with the HBV RNA polymerase protein [37]. Specifically, for the docking of hydroxytyrosol with HBV polymerase, the docking grid was centered at the coordinates (X = 6.841, Y = 2.412, and Z = 21.638) with dimensions set to 66 × 68 × 58. For the docking of lamivudine with HBV polymerase, the docking grid was centered at the coordinates (X = 6.841, Y = 2.412, and Z = 21.638) with dimensions set to 66 × 66 × 63. Finally, the optimal conformation was selected and mapped with pymol software version 2.6.

### 4.10. Statistical Processing

Statistical analysis of data was performed using SPSS 25.0 software. Data mapping was performed using GraphPad Prism 9, and differences were compared using ($\bar{x}$ ± s). * *p* < 0.05 indicates statistically difference. ** *p* < 0.01 indicates statistically significant results.

## 5. Conclusions

In this study, it was confirmed that hydroxytyrosol isolated from Lindernia Ruellioides has low drug toxicity and obvious anti-HBV activity in the appropriate concentration range and has a good inhibitory effect on HBsAg, HBeAg, and DNA in HepG2.2.15 cells. Molecular docking analysis showed that hydroxy-tyrosol has strong interaction with HBV polymerase. It is possible to inhibit HBV polymerase to reduce the secretion of antigen and block the transcription of viral genome. However, the mechanism of action needs further experiments.

## Data Availability

Data are available upon request.

## Supplementary Materials

The following supporting information can be downloaded at: https://www.mdpi.com/article/10.3390/molecules30092063/s1. The following content can be obtained from the Supplementary Materials. Figure S1: ^1^H-NMR spectrum of hydroxytyrosol (DMSO-d6, 500 MHz); Figure S2: ^13^C-NMR spectrum of hydroxytyrosol (DMSO-d6, 125 MHz).
