# Supplementary material for: In Vitro Anti-Hepatitis B Virus Activity of Hydroxytyrosol from Lindernia ruellioides
Source: Molecules. 2025 May 6;30(9):2063. doi: 10.3390/molecules30092063 (PMC12073499; doi:10.3390/molecules30092063)
Supplement: Supplementary file 1 [file molecules-30-02063-s001.zip › molecules-3541958-supplementary.pdf]

Supplementary Material

In Vitro Anti-Hepatitis B Virus Activity of Hydroxytyrosol from *Lindernia ruellioides*

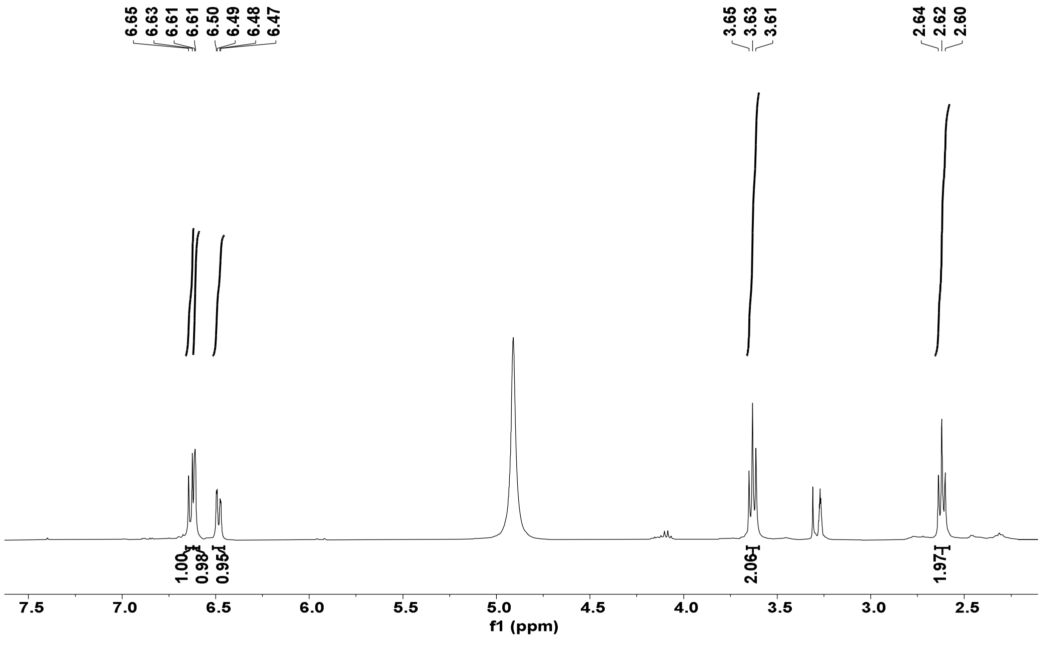

Figure S1: ^{1}H-NMR of hydroxytyrosol.

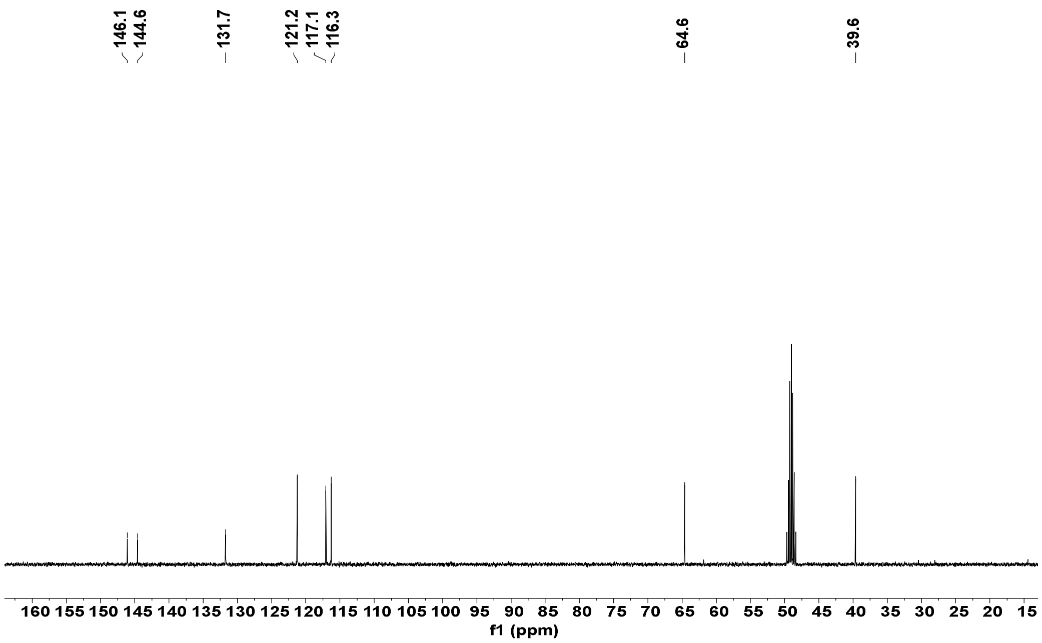

Figure S2: ^{13}C-NMR of hydroxytyrosol.
